# A serum metabolomics study of patients with nAMD in response to anti-VEGF therapy

**DOI:** 10.1038/s41598-020-58346-3

**Published:** 2020-01-28

**Authors:** Yan Gao, Yi Chong Kelvin Teo, Roger W. Beuerman, Tien Yin Wong, Lei Zhou, Chui Ming Gemmy Cheung

**Affiliations:** 1 Singapore Eye Research Institute, Singapore, Singapore; 2 Singapore National Eye Centre, Singapore, Singapore; 3 Department of Ophthalmology, Yong Loo Lin School of Medicine, National University of Singapore, Singapore, Singapore; 4 Ophthalmology and Visual Sciences Academic Clinical Research Program, Duke-NUS Medical School, National University of Singapore, Singapore, Singapore

**Keywords:** Metabolomics, Predictive markers, Macular degeneration

## Abstract

Intravitreal injection of anti-vascular endothelial growth factor (anti-VEGF) is the current standard of treatment for choroidal neovascularization (CNV) secondary to neovascular age-related macular degeneration (nAMD), but there are no diagnostic tools to predict response of these therapies. We hypothesize that differences in baseline metabolic profiles of patients with nAMD may influence responsiveness to anti-VEGF therapy, and thus provide prognosticating information for these patients. A prospective study was performed on 100 patients with nAMD treated with anti-VEGF therapy. We classified patients into two groups: responders (n = 54) and non-responders (n = 46). The expression levels of glycerophosphocholine,LysoPC (18:2) and PS (18:0/20:4) were higher in non-responders and these findings were verified in the validation cohort, implicating that reductions in these three metabolites can be used as predictors for responsiveness to anti-VEGF therapy during the initial loading phase for patients with nAMD. Our study also provided new insights into the pathophysiological changes and molecular mechanism of anti- VEGF therapy for nAMD patients.

## Introduction

Age-related macular degeneration (AMD) is a common cause of blindness in elderly people^1,2^. The neovascular form of AMD (nAMD) is characterized by abnormal vessel leakage and/or bleeding resulting in the formation of fibrovascular tissue which leads to poor vision without treatment. Intravitreal injection of anti-vascular endothelial growth factor (anti-VEGF) is the current standard of treatment for nAMD, showing excellent visual acuity gains in large pivotal randomized controlled trials^1,3,4^.

However, there remains a broad range of responses to anti-VEGF treatment despite its overall efficacy in the majority of patients. It has been suggested that exudation remains detectable in the eyes of >50% patients after initial 3 months of treatment of anti-VEGF therapy^5^. Current methods used to identify “good” and “poor” responders include stratifying disease status by markers of structure or function using tools such as optical coherence tomography (OCT)^6^, fluorescein angiography for lesion type^7^, and visual acuity tests^8^. Some imaging biomarkers such as the presence of intra retina fluid^9–11^ and clinical signs such as poor starting vision^12,13^ are associated with long-term poor prognosis, but these biomarkers do not precisely predict response to anti-VEGF treatment.

Patients with nAMD have been known to have systemic risk factors that are different from age-matched controls, suggesting generalized alterations^14^. Metabolomics, the global quantitative assessment of endogenous metabolites within a biological system^15^, may identify systemic metabolites responsible for differentiation between individuals despite intra-individual variations^16^. This method could provide metabolite information from environmental and lifestyle factors as well as individual characteristics such as dietary response and disease history^17^. The metabolic profiling of a biological system can reflect the phenotype of the study subject and provide information that is complementary to genomics, transcriptomics or proteomics studies^18^. The aim of our current study is to examine baseline serum metabolic profile in patients with nAMD and to relate this to the anatomical response from anti-VEGF therapy during the initial treatment phase over 3 months (typically referred to as the “loading” dose phase).

## Results

Baseline, month 3 and month 12 characteristics of responders and non-responders are summarized in Table 1. Samples were assigned to training set and validation set. There was no significant difference in the age, gender, or proportion with ischemic heart disease, stroke, diabetes, hyperlipidemia, hypertension, chronic kidney disease or smoking (Table 1).

**Table 1:** Comparison of characteristics of responders and non-responders at baseline and after 3 monthly administrations of anti-vascular endothelial growth factor (VEGF) therapy.

	Testing set			Validation set		
	Responder	Non-responder	p-value	Responder	Non-responder	p-value
Eyes, n	29	21	—	25	25	—
Age, years, mean (CI)	73.3 (69.8–76.8)	73.7 (69.2–78.2)	0.89	72.2 (68.6–75.8)	70.7 (67.3–74.1)	0.54
Sex, male, n, (%)	15 (51.7)	13 (61.9)	0.48	16 (64.0)	13 (52.0)	0.4
**Systemic conditions at baseline**						
IHD, n, (%)	3 (10.3)	0 (0.0)	0.13	2 (8.0)	3 (12.0)	0.65
Stroke, n, (%)	4 (13.8)	3 (14.3)	0.96	1 (4.0)	0 (0)	0.32
Diabetes, n, (%)	11 (37.9)	3 (14.3)	0.07	9 (36.0)	6 (24.0)	0.36
Hyperlipidaemia, n, (%)	18 (62.1)	13 (61.9)	0.99	18 (72.0)	15 (60.0)	0.38
Hypertension, n, (%)	20 (68.9)	13 (61.9)	0.61	15 (60.0)	20 (80.0)	0.13
Smoking, n, (%)	3 (10.3)	4 (19.0)	0.39	6 (24.0)	7 (28.0)	0.75
Chronic kidney disease, n (%)	0 (0)	0 (0)	—	0 (0)	0 (0)	—
**Clinical characteristics**						
VA at baseline, logMAR units, (CI)	0.89 (0.68–1.10)	0.88 (0.64–1.12)	0.94	0.89 (0.66–1.12)	0.63 (0.48–0.78)	0.07
VA at month 3, logMAR units, (CI)	0.60 (0.43–0.77)	0.71 (0.50–0.92)	0.32	0.55 (0.41–0.82)	0.65 (0.45–0.88)	0.71
VA at month 12, logMAR units, (CI)	0.50 (0.28–0.74)	0.68 (0.38–0.85)	0.51	0.52 (0.31–0.78)	0.63 (0.31–0.82)	0.52
VA change from baseline to month 3, logMAR units, (CI)	−0.26 (−0.37–−0.08)	−0.15 (−0.31–0.02)	0.31	−0.28 (−0.35 – −0.10)	−0.05 (−0.25–0.01)	0.69
VA change from baseline to month 12, logMAR units, (CI)	−0.38 (−0.45 – −0.12)	−0.14 (−0.30 − 0.02)	0.58	−0.30 (−0.35 – −0.09)	−0.03 (−0.23–0.01)	0.53
CRT at baseline, μm, (CI)	488 (407–569)	488 (402–574)	0.99	426 (367–485)	476 (427–525)	0.21
CRT at month 3, μm, (CI)	291 (252–330)	515 (399–631)	<0.01	275 (257–293)	364 (321–407)	<0.01
**Lesion type, PCV, n (%)**	16 (55.0)	10 (47.6)	0.12	12 (48.0)	13 (52.0)	0.82
**Anti VEGF agent type**						
Bevacizumab, n, (%)	21 (72.4)	20 (95.2)	0.07	25	25	—
Ranibizumab, n. (%)	1 (3.4)	—	—	—	—	—
Aflibercept, n, (%)	7 (24.1)	1 (4.8)	0.11	—	—	—

Abbreviations: IHD, Ischemic heart disease; VA, visual acuity; logMAR, logarithmic of the minimum angle of resolution; CI, confidence interval; CRT, central retinal thickness.

### Clinical characteristics and response after administration of 3 monthly anti-VEGF treatments

There was no difference in baseline visual acuity (VA) (0.89[0.68–1.10] versus 0.88[0.89–1.12], p = 0.94 in training set; 0.89[0.66–1.12] versus 0.63[0.48–0.78], p = 0.07 in validation set) and central retinal thickness (CRT) (488 μm [407–569] versus 488 μm [402–574], p = 0.99 in training set; 426 μm [367–485] versus 476 μm [427–525], p = 0.21 in validation set) between responders and non-responders. At month 3 and month 12 mean VA was better in the responder group compared to non-responders, although the difference was not statistically significant (0.60[0.43–0.77] versus 0.71[0.50–0.92], p = 0.32 at month 3 and 0.50[0.28–0.74] versus 0.68[0.38–0.85], p = 0.51 at month 12 in training set; 0.55[0.41–0.82] versus 0.65[0.45–0.88], p = 0.71 at month 3 and 0.52[0.31–0.78] versus 0.63[0.31–0.82], p = 0.52 at month 12 in validation set). There were more gains in vision at month 3 and month 12 from baseline in responder groups compared to the non-responder groups for both training and validation sets, however this was not statistically significant. Responders had significantly thinner CRT than non-responder at month 3 (291 μm [252–330] versus 515 μm [399–631], p < 0.01 in training set; 275 μm [257–293] versus 364 μm [321–407], p < 0.01 in validation set).

### High-resolution mass spectral data

**Figure 1:**
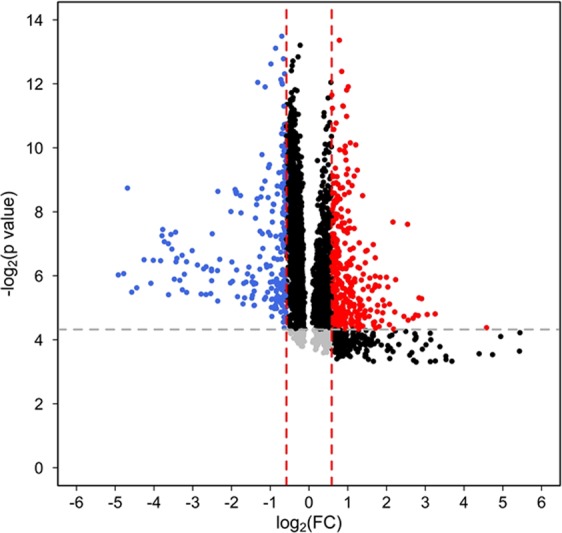
Volcano plot of serum metabolome comparing responders versus non-responders. Cutoff for p value is < 0.05; fold change (nonresponders/responders) cutoff is >1.5 or <0.66.

Mass spectral data extraction from RP positive, RP negative, HILIC positive and HILIC negative modes using XCMS online yielded 3944, 1999, 4827 and 2135 m/z features defined by high-resolution m/z, retention time and ion intensity, respectively. Volcano plot showing p value and fold change cutoff for metabolite features were shown in Fig. 1.

**Figure 2:**
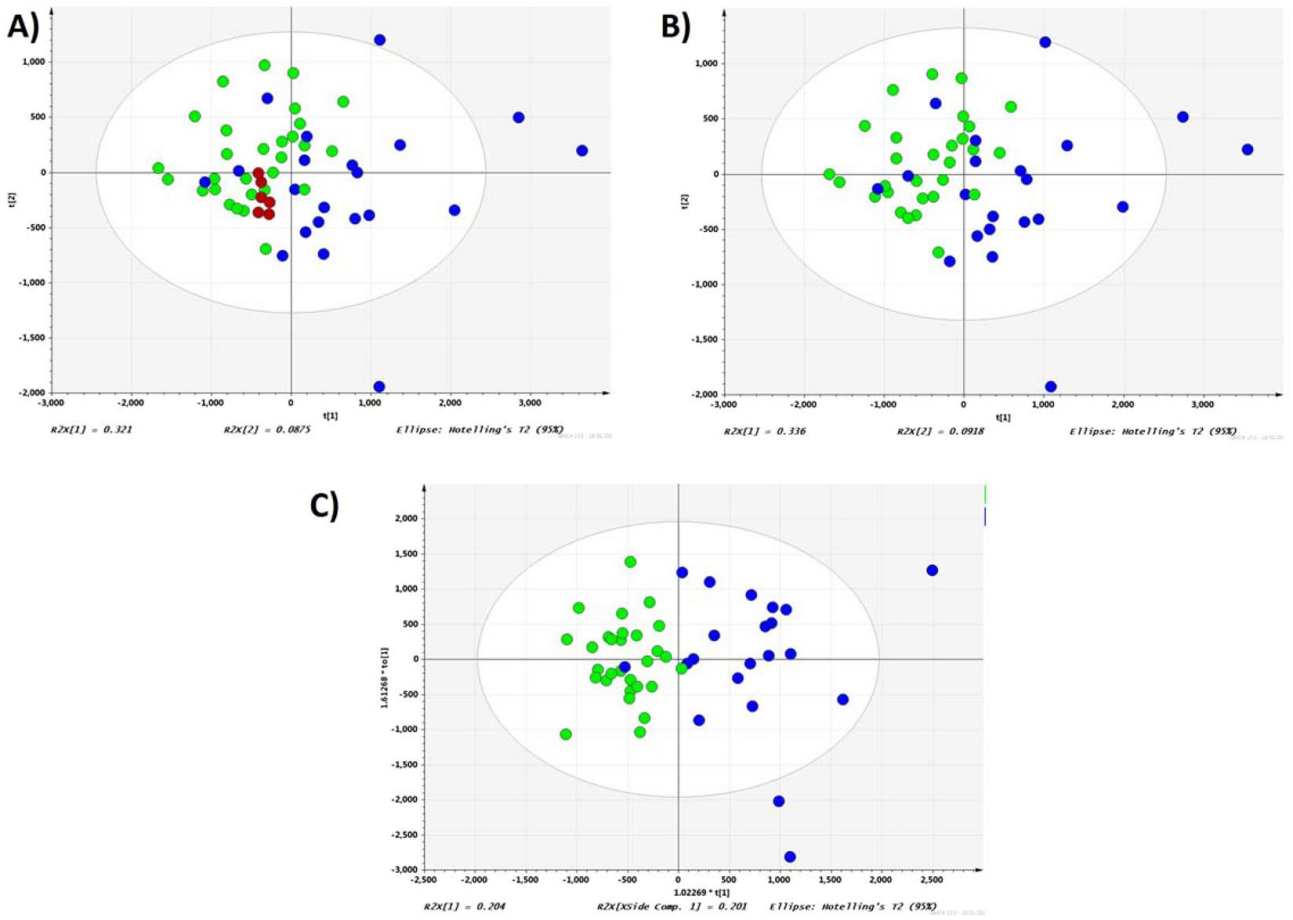
PCA and OPLD-DA score plot of the untargeted metabolomics analysis of serum samples. (**A**) PCA score plot of responders, non-responders and QC samples (R2 = 0.683, Q2 = 0.416); (**B**) PCA score plot of responders and non-responders (R2 = 0.67, Q2 = 0.417); (**C**) OPLS-DA score plot of responders and non-responders (R2 = 0.405, Q2 = 0.378). • -responders; •-non-responders; •- QC.

Principal component analysis (PCA) model constructed from aligned peak data from responders and non-responders in training set was optimized at 7 principal components, with R2 and Q2 value at 0.67 and 0.417, respectively. The first component explained 33.6% of the variance as shown in Fig. 2B. Most samples from responders are located toward the negative scores while non-responders are located toward the positive scores along the first principal component.

Orthogonal projection to latent structures discriminant analysis (OPLS-DA) was used to identify the *m/z* features responsible for the differentiation between nAMD responders and non-responders observed in PCA score plot. After removal of the first orthogonal component (20.1% of variation), the first predictive component (20.4% of variation) could obviously separate responders from non-responders (Fig. 2C, R2 = 0.405, Q2 = 0.378, cross validation analysis of variance [CV-ANOVA], p value < 0.0005). The 999 times permutation test Q2 intercept was −0.394, demonstrating the stability and non-randomness of our model. The score plot of OPLS-DA model showed clear separation between responder group and non-responder group, implicating that this model could explain the differentiation between these two groups. S-plot and variable importance for the projection (VIP) plot were used to identify the *m/z* features responsible for the separation. *m/z* features with high contribution to the variation and correlation within the dataset (top and bottom 10% values of p[1] and p(corr) [1] in S plot and VIP > 1) were selected as potential biomarkers. A list of identified metabolites can be found in Supplementary Table S4.

**Figure 3:**
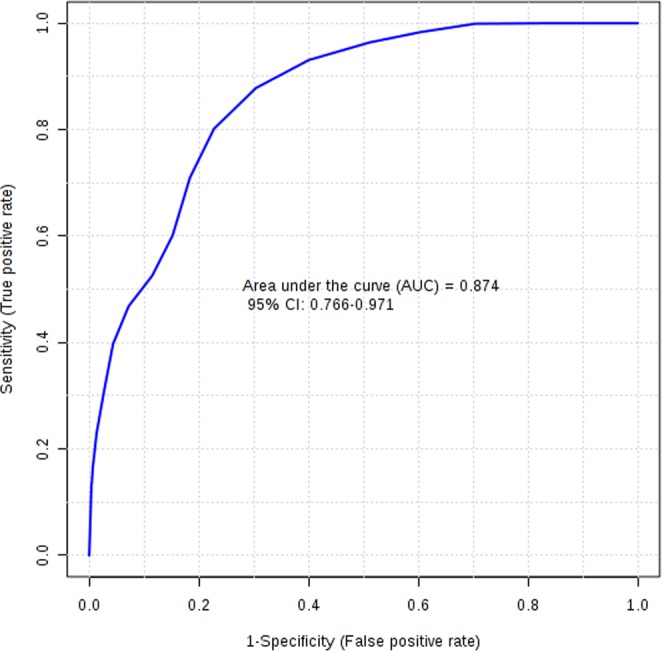
Receiver-operating characteristic curve for validation of metabolomics classification of responders and non-responders.

The general metabolomics signature diagnostic for anti-VEGF responses in patients with nAMD was then subjected to validation in an independent dataset consisting of 25 responders and 25 non-responders. The diagnostic signature had a sensitivity of 66.6% and a specificity of 82.7%. Overall the precision of the model (positive predictive value) was 73.7%. The area under the receiver-operating characteristic (AUROC) was 0.874 (95% CI, 0.766–0.971) (Fig. 3).

### Interpretation of metabolic differences between responders and non-responders

**Figure 4:**
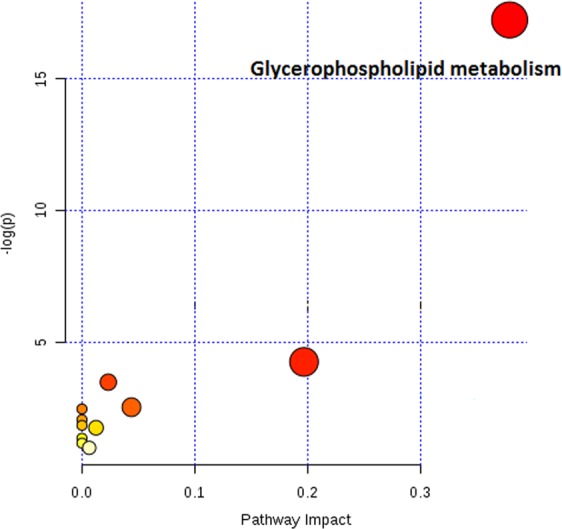
Graph showing pathway analysis based on metabolites associated with differentiation between responders and non-responders of AMD patients. −log(p) = minus logarithm of the p value. The node color is based on its p value and the node radius is determined based on their pathway impact values.

**Figure 5:**
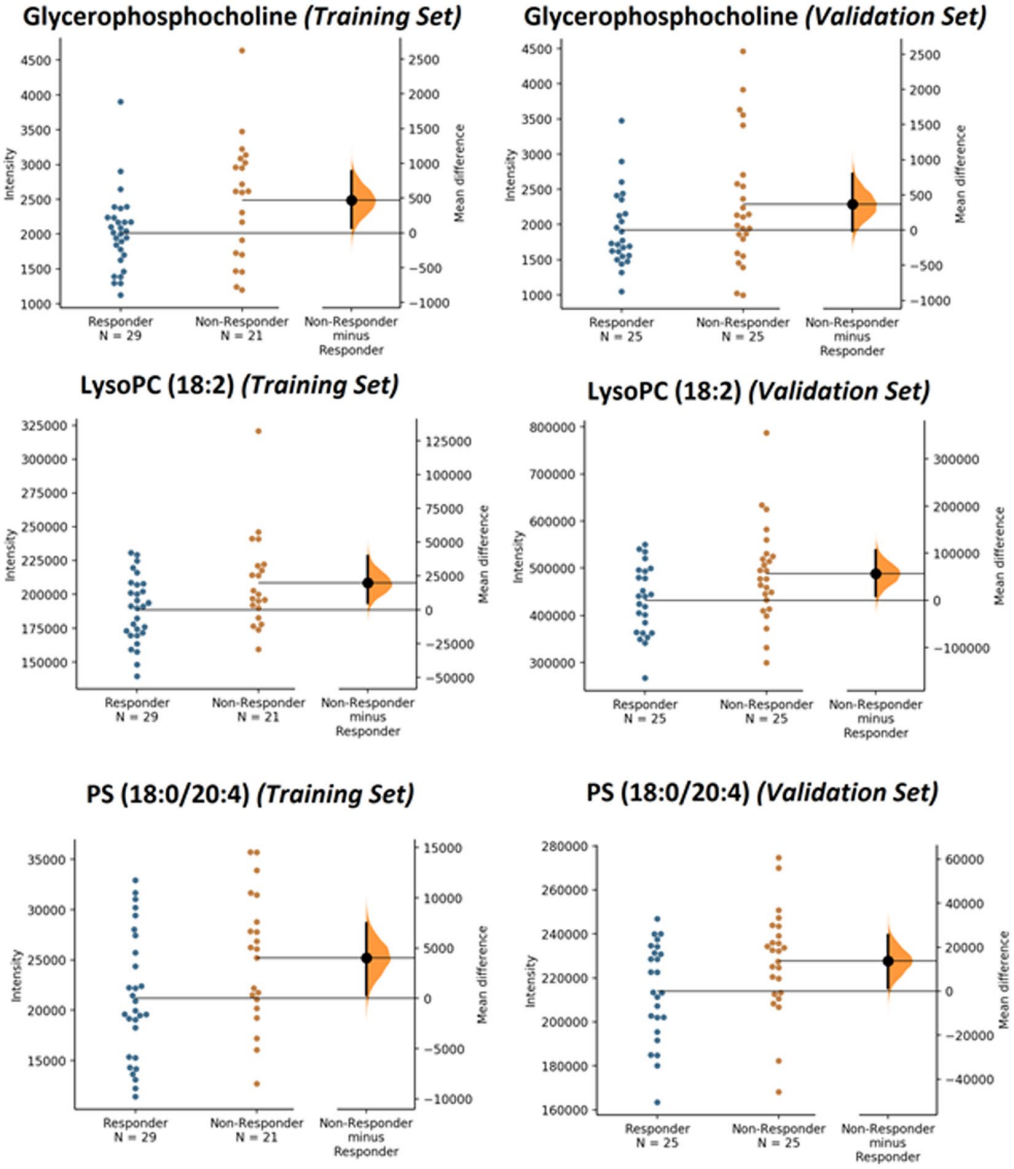
Estimation plots of altered metabolites in responders and non-responders of AMD patients^63^. The mean difference is depicted as a dot and the 95% confidence interval is indicated by the ends of the vertical error bar.

**Figure 6:**
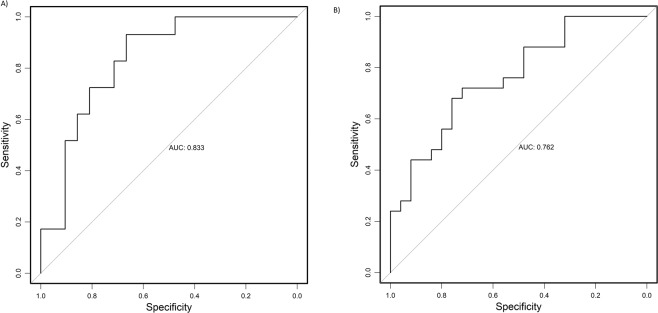
Receiver-operating characteristic curve for three metabolite biomarkers (glycerophosphocholine LysoPC (18:2) and PS (18:0/20:4)) in training set (**A**) and validation set.

An analysis of the LC-MS spectra was conducted to identify which metabolites were contributing to the metabolic profile differentiation between responders and non-responders. Pathway analysis of these identified metabolites revealed glycerophospholipid metabolism alteration (Fig. 4). Compared with profiles from non-responders, serum profiles from responders had significantly lower level of glycerophosphocholine, LysoPC (18:2) and PS (18:0/20:4) in training set (p = 0.023, q = 0.0553; p = 0.020, q = 0.0529; p = 0.032, q = 0.0529). These results were confirmed in the validation set (LysoPC (18:2) p = 0.031, q = 0.0743; PS (18:0/20:4) p = 0.038, q = 0.0743). Similar trend, although not reaching statistical significance was also observed for glycerophosphocholine (p = 0.087, q = 0.1042) (Fig. 5). Glycerophosphocholine was also verified by pure standards (see Supplementary Figure S1). The AUROC for these three metabolites in training set and validation set was 0.833 and 0.762, respectively (Fig. 6).

## Discussion

Previous metabolomics studies have shown patients with nAMD are different in metabolic profiles from similarly aged persons without nAMD in pathways including tyrosine metabolism, sulfur amino acid metabolism, amino acids related to urea metabolism^16^ and enrichment of glycerophospholipid pathway^19,20^. Osborn *et al*. found significant differences in metabolites including peptides, bile acids and vitamin D in patients with nAMD compared to age matched controls, and summarized that tyrosine and urea metabolism may be important in AMD pathophysiology^16^. Another metabolomics study investigating AMD patients revealed that glycerophospholipid pathway is associated with significantly altered metabolites between control group without any vitreoretinal disease and AMD group^19^. Our group has previously found higher serum level of glycerophospholipids, covalently modified amino acids and di/tri-peptides, fatty acids and carnitines in patients with choroidal neovascularization and polypoidal choroidal vasculopathy compared to healthy controls^20^. Small changes were also detected in the levels of some amino acids, organic acids, dimethyl sulfone and specific moieties when investigating the plasma metabolomics profiles of patients with AMD^17^. The intestinal microbiomes of nAMD patients were shown to be enriched in genes of the L-alanine fermentation, glutamate degradation and arginine biosynthesis pathways and decreased in genes of the fatty acid elongation pathways^21^. In this study, we now provide evidence that differences in baseline metabolomics signatures in nAMD patients may also predict their responses to the initial treatment (3 monthly anti-VEGF injections during the “loading phase”).

We found that the serum level of glycerophosphocholine (GPC) was higher in non-responders compared to responders. GPC has been recognized as a degradation product of phosphatidylcholine, which is one of the most important glycerophospholipids in mammalian cells^22^. Increased level of GPC has been detected in cerebrospinal fluid of Alzheimer patients^23^ and a favorable response to neoadjuvant chemotherapy is associated with a reduction in GPC concentration during the treatment for patients with breast cancer^24^. The breakdown of phosphatidylcholine may be reflected in elevated concentrations of GPC in serum and results in altered phosphatidylcholine metabolism^22,25^. A correlation between phosphatidylcholine metabolism and tumor malignancy and angiogenesis has been reported by Baek^26^ and Chen^27^. Higher concentration of GPC in non-responders may be associated with increased angiogenesis that potentially can be used as a predictor of anti-VEGF therapy.

Elevated levels of LysoPC (18:2) and PS (18:0/20:4) were also detected and validated in non-responders. Lysophosphatidylcholine (LysoPC) is a breakdown product of phosphatidylcholine and higher levels of LysoPC have been linked to the cardiovascular complications associated with atherosclerosis^28^, ischemia^29^ and diabetes^30^. LysoPC can be found in cell membrane or the polar surface of oxidized lipoproteins and plays an important role in vascular development^31^.A study by Zou *et al*. revealed that higher level of LysoPC in aged aorta from rats is likely responsible for reactive species generation, and thus enhances oxidative stress in old rat aorta^32^, suggesting that increased level of LysoPC may play a significant role on redox balance during the vascular aging process. LysoPCs are likely to be degraded from glycerophospholipids by the activity of phospholipase enzymes (sPLA_2_)^33^. Glycerophospholipids are important for maintaining structural stability and membrane fluidity and have been implicated in initiation and promulgation of oxidative stress in neurological disorders^34^. Accumulation of LysoPC (18:2) in serum might have damaging effect on vascular modelling by induction of oxidative stress and thus result in poor response to anti-VEGF therapy in our study. Phosphatidylserine (PS) is predominately localized in the inner membrane leaflet and this asymmetry is actively maintained by ATP-dependent lipid transporters regulations^35^. The loss of asymmetric distribution of phospholipid might results in changes of membrane biochemical properties. Dysregulation of PS has been found in tumor microenvironment and antagonizes tumor immunity development by acting as a global immunosuppressive signal in efferocytosis, infectious disease and cancer^36^. Based on these evidences, agents targeting PS could have significant values in cancer and infectious disease therapeutics. Similarly, Li *et al*. reported that PS is exposed in CNV endothelium and thus suggested antibodies targeting exposed PS may have therapeutic value in CNV^37^. Therefore, up-regulation of PS (18:0/20:4) might have side effects on AMD recovery.

Progressive Bruch’s membrane thickening and deposition of extracellular deposits with abundant lysophospholipid and free fatty acids as drusen have been noted on histological sections of eyes with AMD, suggesting the role of phosphatidylcholine hydrolysis as potential pathogenic mechanism in AMD^38–42^. However, the exact role of serum lipid levels in AMD is not yet clear and studies on the association of serum lipid and AMD risk have been inconsistent^43–45^. No significant difference in lipoprotein (a) concentrations was observed between AMD patients with control groups in the study by Nowak *et al*.^46^ and there was no significant difference in total cholesterol, triglycerides, phospholipids, high and low density lipoprotein-cholesterol concentration when compared AMD patients with controls in another study^45^. On the other hand, Reynolds and colleagues revealed that higher total cholesterol and low density lipoprotein were associated with increased risk whereas higher high density lipoprotein levels tended to reduce AMD risk^44^. These controversial results might be due to high variability of lipid and fatty acid levels and the use of medication and/or dietary intake^47^. Chen *et al*. detected elevated serum level of glycerophospholipids in choroidal neovascularization and polypoidal choroidal vasculopathy group compared to healthy controls in an untargeted metabolomics study^20^. Our results further support that phosphatidylcholine hydrolysis may be more prominent in non-responders.

The current study has a number of limitations; firstly, a relatively small sample size was assessed. Secondly, response was determined by anatomical changes after the initial treatment phase (after the first 3 treatments). The anatomical change as assessed on OCT provides the best objective measure of response. Other functional outcomes were also analysed at longer time points (12 months) but did not achieve statistical significance, most likely due to the small sample size or the mismatch between functional and anatomical markers that is often observed in AMD treatment^48^. Lastly, we considered all currently available anti-VEGF agents in this study with a vast majority of patients receiving bevacizumab. This may have affected the proportion of responders versus non-responders.

It will be important to explore whether the findings from this study are reproducible in an independent cohort and thus further testing in other clinical cohorts with nAMD will instruct on the utility of these diagnostic biomarkers for screening. Further exploration into the reproducibility of findings in this study from different ethnic groups should also be considered. It is also of interest to explore how metabolic profiles differentiate among healthy control, responders and non-responders. A metabolomics study of all participating patients after 3 months’ treatment will also provide valuable information to confirm if these metabolite biomarkers are still significantly altered.

## Conclusion

In this study, we investigated serum metabolomics profile for responders and non-responders to anti-VEGF therapy during the initial 3 monthly “loading” phase of treatment among a cohort of nAMD patients, which was validated in an independent dataset. We found increased levels of GPC, LysoPC (18:2) and PS (18:0/20:4) in non-responders, implicating significant impairment to glycerophospholipid metabolism. These biomarkers could be used as predictive responses to initial anti-VEGF therapy. By differentiating responders and non-responders to the current treatment early in patients’ treatment journey, we suggest that such biomarker information may offer an indication to consider an early switch to different agent or class of drug. This is especially relevant now with newer therapies with different pharmacokinetics and modes of action such as brolucizumab and faricimab currently under study^5,49^. Our findings might provide treatment information for AMD patients and offer novel targets for AMD pathogenesis.

## Materials and Methods

### Study design and participants

We performed a prospective case-control study using baseline serum from a total of 100 participants with nAMD who participated in a prospective clinical cohort study, the Asian AMD Phenotyping Study as described previously^50,51^. Briefly, the study prospectively recruited consecutive treatment-naıve participants with nAMD from the retinal clinic of the Singapore National Eye Centre from March 2010 and is still ongoing. The study was approved by the SingHealth Institutional Review Board (IRB Approval number: 2009/788/A) and was conducted in accordance with the Declaration of Helsinki (protocol number R697/47/2009 and R498/47/2006). Informed consent was obtained from all participants.

### Demographic and medical history

Baseline socio-demographic and medical history was collected using an interviewer-administered questionnaire which was previously validated^52–54^. Data included information on participants’ lifestyle factors, history of smoking, current medications, systemic medical and surgical history.

### Clinical measurement variables

At the baseline visit, all patients underwent a full ophthalmic examination, color fundus photography, fluorescein and indocyanine green angiography and optical coherence tomography (OCT) (Heidelberg Engineering GmbH, Dossenheim, Germany). Baseline measure of best corrected visual acuity (VA) recorded as whichever reading was best: uncorrected, corrected or pinhole, was expressed as the logarithm of the minimum angle of resolution (logMAR). Central retinal thickness (CRT) was obtained using the in-built software where an automated segmentation algorithm was used to produce retinal thickness map of the central 1 mm zone.

All patients received three injections at monthly intervals of intravitreal anti-VEGF. The choice of agent type (aflibercept, bevacizumab or ranibizumab) was decided by the treating physician.

Patients were evaluated at month 3 and categorized into treatment responders (responder group, n = 54) or treatment non responders (non-responder group, n = 46). Treatment response was based on OCT findings of disease activity. Responders were defined as eyes with no sub- or intra-retinal fluid at month 3. Non-responders were defined by persistent sub- or intra- retinal fluid at month 3. All OCT scans were qualitatively analysed by 2 graders blinded to each other’s decision (KYCT, CMGC). Any grading disagreement was openly arbitrated and the final decision was made by the senior grader (CMGC).

### LC-MS based metabolic profiling analysis

The recruited samples were randomly divided into two independent cohorts, i.e. a training set and a validation set. The training set, including 29 responders and 21 non-responders, was used to establish if serum metabolomics profiles could distinguish between patients with nAMD regarding their response to anti-VEGF injections. The validation set, comprising 25 responders and 25 non-responders, was used to independently validate the metabolite biomarkers and assess the effect of anti-VEGF on nAMD patients.

After enrolment, blood was extracted from the cubital vein of each participant. The blood was then immediately transferred to the collection tube and kept at room temperature for 30 min to allow clotting. The clotted blood samples were centrifuged at 3000 g at 4 °C for 20 min to eliminate the supernatant serum and then quickly stored at −80 °C prior to metabolomics detection.

Metabolites were extracted from 200 µl serum samples using 800 ul ice cold 1:1:1(v/v/v) methanol/acetone/acetonitrile, incubated at −20 °C for 30 min, and centrifuged at 16,000 g for 15 min (4 °C) to remove protein. Each sample extract was divided into two equal aliquots and dried in a vacuum concentrator before LC-MS analysis.

Each sample was analyzed both on reverse phase (RP) column and hydrophilic interaction chromatography (HILIC) column in positive and negative ionization modes, i.e. RP + , RP-, HILIC + , HILIC- (Table S1). Aliquots for RP injection were reconstituted in 25 µl 2% acetonitrile and aliquots for HILIC column injections were reconstituted in 25 µl 80% acetonitrile. Metabolites separation was performed on an ACQUITY I-class UPLC system (Waters, Milford, Massachusetts, US). The injection volume was 10 µl and flow rate was 0.6 ml/min. The column and auto-sampler were maintained at 40 °C and 10 °C, respectively. Table S1 listed the columns, mobile phases and gradients for RP and HILIC. Quality control samples were prepared by pooling equal volume of all serum samples in this study to monitor the stability and repeatability during LC-MS analysis. The pretreatment of QC samples was the same as that of real samples and were injected after every ten samples.

Mass detection was achieved on a TripleTOF 5600 fitted with a DuoSpray ion source (SCIEX, Foster, California, US). Mass calibration was automatically performed after every 20 injections by the automated calibration delivery system. The source voltage was set to 5500 V for positive ionization and 4500 V for negative ionization mode. The declustering potential was 80 V and source temperature was 500 °C for both polarities. The curtain gas flow, nebulizer and heater gas were set to 30, 55 and 60 arbitrary units, respectively. Information dependent acquisition (IDA) was used to collect full scan MS and MSMS information simultaneous with an m/z mass range of 100–1000. The instrument performed a TOFMS survey with 160 ms accumulation time, followed by 5 MSMS scans with 18 ms accumulation time. The collision energy was linearly ramped from 20 to 40 V. The following parameters were also applied to data acquisition: dynamic background subtraction, charger monitoring to exclude multiple charged ions and dynamic exclusion of former target ions for 1 s.

Peak extraction and quantification of ion intensities were performed using both XCMS online^55^ and Markerview (SCIEX), which provide lists containing m/z values, retention time and integrated ion intensity for each m/z features.

### Statistical analysis

Descriptive data are presented as mean (confidence interval) or number (percentage). Statistical tests such as Student’s t-test, and chi squared test were used where appropriate to compare demographic and clinical characteristics between the responder and non-responder groups. Analyses for demographic and clinical characteristics were calculated using R V3.3.1^56^.

A combination of analysis of the variance (ANOVA) and multivariate analysis methods including principle component analysis (PCA) and orthogonal partial least squares-discriminant analysis (OPLS-DA) using SMICA (Umertrics, Umea, Sweden) were used to select potential metabolites which are the most responsible for the differentiation between groups. Student’s t-test was used for statistical comparison of pairs of groups and a p value < 0.05 and q value < 0.1 (adjusted using logistic regression) was considered as a priori to be statically significant. The peak lists from both positive and negative mode were normalized by total ion intensity and Pareto scaled first. A PCA was first performed to show a trend of intergroup separation on the score plots. The tight cluster of QC samples in PCA score plot indicated robustness of our metabolic profiling platform (Fig. 2A). R2Y and Q2Y scores were used for assessment of variance coverage by predictive component and model predictability in a seven times cross-validation, respectively^57^. A 999 times permutation test was carried out to confirm the stability and robustness of OPLS-DA model. A Q2 intercept of zero or below from permutation test demonstrates the stability and non-randomness of the model and thus strongly supports the validity of the model^58^.

### Metabolite annotation and pathway analysis

Metabolites identification was achieved by database search against accurate m/z and MS/MS spectra with METLIN^59^ and HMDB^60^. MetaboAnalyst was used for pathway analysis^61^. Selected metabolites were further validated by commercially available pure standards. GPC was purchased from Sigma-Aldrich (St. Louis, Missouri, US).

## Supplementary information

Supplementary table 1–3.

## Data Availability

All the metabolomics datasets described in our study can be accessed at MetaboLights^62^ (https://www.ebi.ac.uk/metabolights/) (Project ID: MTBLS950). All other data supporting the findings of this study are included in this published article as Supplementary Data.
